# The factors and outcomes of stigma toward mental disorders among medical and nursing students: a cross-sectional study

**DOI:** 10.1186/s12888-022-03996-y

**Published:** 2022-05-25

**Authors:** Na Meng, Xia Huang, Jingjun Wang, Mengmeng Wang, Ya Wang

**Affiliations:** 1 Mental Health Center, West China Hospital, Sichuan University / West China School of Nursing, Sichuan University, Chengdu, China; 2 West China School of Nursing, Sichuan University / West China Hospital, Sichuan University, Chengdu, China; 3 School of Nursing, Peking University, Beijing, China; 4 Department of Nursing, West China Hospital, Sichuan University / West China School of Nursing, Sichuan University, No.37 Guoxuexiang, Chengdu, China

**Keywords:** Stigma, Medical students, Mental disorders

## Abstract

**Background:**

Medical and nursing students’ attitudes toward mental disorders have a large impact on their working intentions in mental health settings and patients’ health outcomes. However, there are few studies about the stigma toward mental disorders among medical and nursing students in China.

**Methods:**

In this cross-sectional study, a total of 838 medical and nursing students completed questionnaires on their sociodemographic characteristics and familiarity with people diagnosed with mental disorders as well as the Community Attitudes toward Mental Illness Scale (CAMI). The stigma was compared between medical students and nursing students by ANOVA. A multiple logistic regression model was built to explore the relationships among sociodemographic characteristics, familiarity with mental disorders and stigma.

**Results:**

The total mean score of the CAMI was 137.61 (SD = 15.63). The score for authoritarianism (M = 33.33, SD = 3.62) was the lowest score of the four subscales. Medical students showed more positive attitudes toward mental disorders than nursing students. However, after controlling the co-variables, the difference disappeared. Stigma was significantly associated with students’ education, area of residence, marital status, economic status, history of mental disorders and familiarity with mental disorders.

**Conclusions:**

Medical and nursing students show a negative attitude toward mental illness to a certain degree, especially regarding the view that people with mental disorders are inferior. Higher education level, residence in urban areas, single marital status, better economic status, and better familiarity with mental disorders may be related to less stigma among medical and nursing students.

## Background

Currently, mental disorders are the most prevalent diseases around the world. More than 1 billion people (16% of the world’s population) are affected by mental and addictive disorders in 2016 [1]. In Japan, 22% of people suffer from any common mental disorder during their lifetime [2]. In China, the weighted 12-month and lifetime prevalence of any mental disorder (excluding dementia) are 9.3 and 16.6%, respectively [3]. However, it is quite common for people to hold negative perceptions toward mental disorders and people with mental disorders. People with mental disorders experience a lack of comprehension from society at large [4], as well as avoidance and discrimination [5]. As the core notion in the topic of negative perceptions toward mental disorders, stigma is a mark of shame, disgrace or disapproval that leads to rejection, discrimination and avoidance.

Stigma consists of public stigma, perceived social stigma and self-stigma. Public stigma refers to public reactions to mental disorders or people with mental disorders [6]. Perceived social stigma refers to a person’s belief that society holds prejudicial beliefs toward him or her [7]. Self-stigma refers to the reactions of people toward themselves or their mental disorders [6]. Public stigma may result in people with mental disorders developing self-stigma [8]. Perceived stigma could be internalized to promote self-stigma [9].

Public stigma has been widely discussed. However, during the past 20 years, public stigma has not improved with the increased knowledge about mental disorders [10]. Stigma was reported in the healthcare providers even though they may gain more knowledge than the general population [11]. A previous study pointed out stigma would better be addressed in the early years of professional learning [12]. However, stigmatization of people with mental disorders was common in early career professionals [12]. A worldwide online survey across 65 countries showed that stigma toward both psychiatry and psychiatric patients was common among undergraduate medical students [13]. Medical students and nursing students believed that people with mental disorders symbolized aggression as well as unpredictability, and would never be able to recover sufficiently [14]. Furthermore, medical and nursing students would like to evaluate working in a psychiatric department job as stressful, overwhelming, emotionally and exhausting, even daunting [15, 16].

Stigma toward mental disorders and patients with mental disorders among medical and nursing students would result in bad outcomes for patients as well as the mental health profession. Medical and nursing students’ stigma toward mental disorders may have an indirect impact on patients’ self-stigma, which decreased the help-seeking behaviors from mental health professionals [17]. Moreover, stigma toward mental disorders was negatively associated with medical and nursing students’ intentions of working in mental health settings [15, 18], which may increase the lack of psychiatrists and psychiatric nurses. Furthermore, medical students and nursing students were two important groups among future healthcare providers to treat patients with mental disorders and to shape medical and nursing professional attitudes [19]. Therefore, medical students and nursing students are important groups for mental health stigma studies. In addition, the stigma may have many variations across cultures, which suggests that the stigma of mental disorders needs to be explored within different cultures [20]. However, there are few studies about the stigma toward mental disorders among medical and nursing students in China. Therefore, it is essential to explore the stigma toward mental disorders among medical and nursing students in China.

Previous studies have explored the factors of stigma in order to improve stigma toward mental disorders in medical and nursing students. A study pointed out that fourth-year nursing students, who already had a clinical internship in the department of psychiatry, were less likely to show stigma than other nursing students [21]. A study found that women showed less stigmatization than men among medical students [22]. However, another study did not observe a correlation between stigma and gender [23]. Masedo [14] pointed out that stigma was not significantly different between medical students and nursing students. However, one study found that medical students showed more stigma toward patients with self-harm than nursing students [24]. Besides sociodemographic factors, the familiarity with mental disorders or people with mental disorders is also related to stigma. Students at a US medical school owning personal experiences with mental illness reported more social acceptance [25]. Similarly, less stigma was reported in nursing students with direct experience with patients diagnosed with mental disorders [26]. In summary, considering several inconsistent results and different cultures, factors of stigma toward mental disorders and patients need to be further verified in Chinese medical and nursing students.

Therefore, we conducted a cross-sectional study to explore the outcomes and factors of stigma toward mental disorders or people with mental disorders among Chinese medical and nursing students.

## Methods

### Participants

For this observational study, 838 students were recruited by convenience sampling from 18th May 2020 to 24th May 2020 in China. Participants met the following criteria: (a) were full-time college students; (b) were majoring in nursing or medicine; and (c) participated voluntarily.

### Measures

#### Sociodemographic characteristics

A self-administered questionnaire was established to collect sociodemographic data, including gender, age, marital status, highest educational level, major, economic status, and area of residence.

### Familiarity with people with mental disorders

Two dichotomous (yes or no) questions were used to collect participants’ familiarity with people with mental disorders. Question 1: Have you suffered from mental disorders? Question 2: Do you have a family member, a relative or a friend experiencing mental disorders?

### Community attitudes toward mental illness scale (CAMI)

The CAMI, a 40-item scale, was developed by Taylor et al. [27]. The scale is widely used to assess public attitudes toward mentally ill individuals. The scale spans four domains: authoritarianism, benevolence, social restrictiveness, and community mental health ideology. Authoritarianism refers to a view that people with mental disorders are inferior to people without mental disorders; according to this view, people with mental disorders require a coercive approach [28]. Benevolence refers to a sympathetic view of people suffering from mental disorders [28]. Social restrictiveness is the view that as a threat to society, people with mental disorders should be avoided [28]. Community mental health ideology refers to the acceptance of community-based care for people with mental disorders [28]. Each item is scored on a scale from 1 (strongly agree) to 5 (strongly disagree). The total score is calculated by adding scores of all items. A higher score indicates more positive attitudes toward people with mental disorders. The Cronbach’s alpha coefficients of the four dimensions were 0.68–0.88 [27]. In our study, the Cronbach’s alpha coefficient of the CAMI was 0.90, and the values for the four dimensions ranged from 0.49–0.77.

### Data collection

The researchers input the questionnaires in WJX, an online survey platform. Then, the questionnaire link was sent to college students individually via their WeChat accounts (a social software similar to WhatsApp). Students could voluntarily participate in the survey. Meanwhile, students were encouraged to share the link with their schoolmates. To avoid invalid repeats, the questionnaires could be completed only once by each WeChat ID. To increase the completeness of questionnaires, students could return to continue to fill out the questionnaire even if they exited the link. Moreover, key features such as the requirement for “full-time college students only” were highlighted in bold red font to avoid invalid responses. This study was approved by the Ethics Committee of one Tertiary Hospital. Online informed consent was obtained from all participants. The information of all participants was anonymous, and each individual had the choice to withdraw from the study at any time.

### Data analysis

Sociodemographics and familiarity with people with mental disorders are expressed as frequencies, percentages (n%), and means ± standard deviations (M ± SD). Scores of the CAMI are described as means ± standard deviations (M ± SD). The CAMI score was compared among groups with different sociodemographic characteristics by ANOVA. The sociodemographic characteristics and stigma were compared between medical students and nursing students by and Chi-square test and ANOVA. A multiple linear stepwise regression model was built to explore the factors associated with stigma among medical and nursing students. The independent variables were listed in Table 1. The statistical significance level was set at *P* < 0.05. All statistical analyses were performed in SPSS version 22.

**Table 1:** Sociodemographic characteristics of medical students (*n* = 838)

Variables	n (%)	CAMI(M ± SD)	F	P
Age ^a^	20.25 ± 2.65	–	–	–
Gender				
Male	85 (10.1)	139.00 ± 18.32	0.74	0.39
Female	753 (89.9)	137.46 ± 15.30		
Marital status				
Married	22 (2.6)	136.36 ± 12.92	0.15	0.70
Single	816 (97.4)	137.65 ± 15.70		
Education				
Junior college or below	644 (76.8)	135.25 ± 14.60	68.83	< 0.001
College or above	194 (23.2)	145.46 ± 16.40		
Domicile				
Urban	497 (59.3)	140.01 ± 16.52	29.62	< 0.001
Rural	341 (40.7)	134.14 ± 13.51		
Major				
Medicine	117 (14.0)	145.47 ± 18.36	35.78	< 0.001
Nursing	721 (86.0)	136.34 ± 14.76		
Economic status / Monthly household income				
Very poor (1500-2999RMB)	57 (6.8)	129.98 ± 12.90	5.98	< 0.001
Relatively poor (3000-4999RMB)	189 (22.6)	136.50 ± 14.30		
General (5000-9999RMB)	535 (63.8)	138.43 ± 15.78		
Relatively well-off (10,000 -14999RMB)	55 (6.6)	140.47 ± 17.89		
Very well-off (≥15000RMB)	2 (0.2)	163.50 ± 27.58		
Have you suffered from mental disorders?				
Yes	6 (0.7)	156.5 ± 17.312	8.91	0.003
No	832 (99.3)	137.48 ± 15.54		
? Do you have a family member, a relative or a friend experiencing mental disorders?				
Yes	142 (16.9)	141.77 ± 15.52	12.23	< 0.001
No	696 (83.1)	136.77 ± 15.53		

*CAMI* community attitude toward the mentally illness scale, *M* mean, *SD* standard deviation, − not Applicable

^a^ mean ± standard deviation

## Results

Students’ sociodemographic characteristics are shown in Table 1. The mean age of the participants was 20.25 years old (SD = 2.65). Approximately 89.9% (*n* = 753) of the participants were female. Nursing students (*n* = 721) accounted for 86% of the participants. Table 1 also shows that the majority of the participants did not suffer from mental disorders (*n* = 832, 99.3%) and did not have a family member, a relative or a friend experiencing mental disorders (*n* = 696, 83.1%). Moreover, the comparison of stigma among different sociodemographic groups is shown in Table 1. Stigma was significantly associated with education level, area of residence, major, economic status, the familiarity with mental disorders (Table 1).

Table 2 shows the scores of the CAMI and subscales. The total mean CAMI score was 137.61 (SD = 15.63). The benevolence score (M = 36, SD = 4.88) was the highest of the four subscales. The score for authoritarianism (M = 33.33, SD = 3.62) was the lowest score of the four subscales.

**Table 2:** The outcome of CAMI

Scale	Total score (M ± SD)	Item score (M ± SD)
Authoritarianism	33.33 ± 3.62	3.33 ± 0.36
Benevolence	36.00 ± 4.88	3.60 ± 0.49
Social Restrictiveness	34.67 ± 4.83	3.47 ± 0.48
Community Mental Health Ideology	33.60 ± 4.45	3.36 ± 0.44
CAMI	137.61 ± 15.63	3.44 ± 0.39

*CAMI* community attitude toward the mentally illness scale, *M* mean, *SD* standard deviation

Table 3 presents the comparison of sociodemographic characteristics and familiarity with mental disorders between medical students and nursing students. Compared with nursing students, medical students would be likely to report male, higher education level, living in urban areas, better economic status and more familiarity with people with mental disorders. The scores of CAMI and subscales were significantly higher in medical students than in nursing students (Table 4).

**Table 3:** The comparison of sociodemographic characteristics and familiarity with people with mental disorders between medical students and nursing students (*n* = 838)

Variables	Medical students *n* (%)	Nursing students *n* (%)	F/χ^2^	P
Age ^a^	19.91 ± 2.42	20.30 ± 2.68	2.16	0.14
Gender				
Male	25 (21.4)	60 (8.3)	18.80	< 0.001
Female	92 (78.6)	661 (91.7)		
Marital status				
Married	2 (1.7)	20 (2.8)	0.45	0.76
Single	115 (98.3)	701 (97.2)		
Education				
Junior college or below	26 (22.2)	618 (85.7)	228.10	< 0.001
College or above	91 (77.8)	103 (14.3)		
Domicile				
Urban	97 (82.9)	400 (55.5)	31.38	< 0.001
Rural	20 (17.1)	321 (44.5)		
Economic status / Monthly household income				
Very poor (1500-2999RMB)	3 (2.6)	54 (7.5)	42.83	< 0.001
Relatively poor (3000-4999RMB)	25 (21.4)	164 (22.7)		
General (5000-9999RMB)	65 (55.6)	470 (65.2)		
Relatively well-off (10,000 -14999RMB)	23 (19.7)	32 (4.4)		
Very well-off (≥15000RMB)	1 (0.9)	1 (0.1)		
Have you suffered from mental disorders?				
Yes	3 (2.6)	3 (0.4)	6.53	0.01
No	114 (97.7)	718 (99.6)		
Do you have a family member, a relative or a friend experiencing mental disorders?				
Yes	28 (23.9)	114 (15.8)	4.72	0.03
No	89 (76.1)	607 (84.2)		

^a^ mean ± standard deviation

**Table 4:** The comparison of scores of CAMI and subscales between medical students and nursing student

Scale	Medical students (M ± SD)	Nursing students (M ± SD)	F	P
Authoritarianism	35.60 ± 4.35	32.97 ± 4.35	56.44	< 0.001
Benevolence	37.86 ± 5.26	35.71 ± 4.75	20.08	< 0.001
Social Restrictiveness	36.60 ± 5.54	34.35 ± 4.63	22.32	< 0.001
Community Mental Health Ideology	35.41 ± 5.49	33.31 ± 4.18	23.08	< 0.001
CAMI	145.47 ± 18.36	136.34 ± 14.76	35.78	< 0.001

*M* mean, *SD*, standard deviation

Table 5 presents the significant outcomes of the linear stepwise regression model. Compared with junior college or below students, college or above students showed a significantly higher CAMI score (B = 8.58, β = 0.23, *P* < 0.001). Compared with urban students, rural students showed a significantly lower CAMI score (B = − 3.41, β = − 0.11, *P* = 0.002). Compared with married students, single students had a higher CAMI score (B = 8.94, β = 0.09, *P* = 0.007). The CAMI score was positively related to economic status (B = 1.87, β = 0.08, *P* = 0.01). Compared with students who suffered from mental disorders, students who did not presented a lower CAMI score (B = -15.53, β = − 0.08, *P* = 0.01). Compared with students who had a family member, a relative or a friend experiencing mental disorders, students who did not showed a lower CAMI score (B = -3.19, β = − 0.08, *P* = 0.02).

**Table 5:** Multiple linear regression analysis with CAMI as dependent variable (*n* = 838)

Variables	B	SE	β	T	P	95%CI
Constant	145.96	14.62		9.98	< 0.001	[117.23, 174.63]
Education level ^a^	8.58	1.33	0.23	6.43	< 0.001	[5.96, 11.20]
Domicile ^b^	-3.41	1.11	−0.11	−3.07	0.002	[−5.59, −1.23]
Marital status ^c^	8.94	3.30	0.09	2.71	0.007	[2.46, 15.41]
Economic status	1.87	0.76	0.08	2.46	0.01	[0.38. 3.35]
Have you suffered from mental disorders? ^d^	− 15.53	6.11	−0.08	−2.54	0.01	[−27.51, −3.54]
Do you have a family member, a relative or a friend experienced mental disorders?? ^d^	−3.19	1.39	−0.08	−2.29	0.02	[−5.92, −0.46]

R^2^ = 0.115, R^2^ adj = 0.109

*CAMI* community attitude toward the mentally illness scale

Reference: ^a^ junior college or below; ^b^ urban; ^c^ married; ^d^ yes

## Discussion

In this study, we aimed to explore the outcomes and factors of stigma toward mental disorders or people with mental disorders in Chinese medical and nursing students. Overall, medical and nursing students showed stigma toward mental disorders or people with mental disorders. We found that stigma was significantly associated with the student’s education, area of residence, marital status, economic status, and familiarity with mental disorders.

The study revealed that medical and nursing students experienced stigma toward mental disorders or people with mental disorders. Similar to a previous study, medical students held a somewhat negative attitude toward mental disorders [28]. In the study, authoritarianism was the lowest-scoring subscale of the CAMI. Authoritarianism refers to the view that people with mental disorders are inferior to people without mental disorders. The results suggested that medical students believe that people with mental disorders are inferior. A previous study also confirmed that 43.8% of the students agreed that one of the main causes of mental illness is a lack of self-discipline and willpower [29]. The benevolence score was the highest-scoring subscale of the CAMI (M = 3.93, SD = 0.94), which suggested that most students could hold a sympathetic view toward people with mental disorders. In total, stigma toward mental disorders is high among medical and nursing students. In addition to the arranged psychological and psychiatric curriculums, schools could organize seminars about stigma and play short videos about stigma to decrease students’ stigma toward mental illness [30]. In the curriculums, teachers could organize role-play sessions focused on interviewing a “psychotic patient” and a “depressed patient” to improve medical and nursing students’ attitudes toward people with mental disorders [31].

In the study, medical and nursing students showed a more positive attitude toward mental disorders than nursing students. The result is not consistent with previous studies. Masedo [14] pointed out that stigma was not significantly different between medical students and nursing students. The possible reason for the discrepancy is that all students were in their last years of the university program in the Masedo’s [14] study and they may get the similar education level. However, in our study, medical students reported significantly a higher education level than nursing students. Another study found that medical students showed more stigma toward patients with self-harm than nursing students [24]. The inconsistent result may result from medical students who reported being less familiar with self-harm than nursing students [24]. In contrast, medical students reported more familiarity with people diagnosed with mental disorders in our study. According to the regression model, stigma has no significant difference between medical students and nursing students with the control of co-variables, such as education level and familiarity with mental disorders. Therefore, we would like to suggest that we need to pay more attention to the students with a lower education level and less familiarity with mental disorders rather than their specialties.

The study confirmed the relationship between stigma and an individual’s socioeconomic status. In the past literature, researchers have usually considered education and income as indicators of socioeconomic status [32, 33]. First, the study revealed that college students showed more positive attitudes toward mental disorders than students in a junior college or below. This result is consistent with another study showing that people with higher education showed a more favorable attitude toward people with mental disorders [33]. In contrast, one study pointed out that years of medical education was not significantly associated with medical students’ attitudes toward mental illness [25]. This result may be due to a ceiling effect. In the first year of training, students show a positive attitude in social acceptance [25]. Hence, in Chiles’s study, education level was not a factor of stigma. In addition, better economic status was related to less stigma toward mental disorders. This result was similar to Letovancova’s [34] result that socioeconomic status influenced attitudes toward people with mental illness. Overall, students with better socioeconomic status showed more positive attitudes toward mental disorders. A possible reason is that higher socioeconomic status means higher health literacy [35]. For students with a low education level and low income, digital video interventions as an understandable and accessible way could enhance mental health literacy and increase positive attitudes [36].

The study also revealed that urban medical and nursing students hold more positive attitudes toward mental disorders than rural medical and nursing students. Another study also pointed out that the level of stigma toward mental illness was significantly higher in rural areas than in urban areas [37]. Ndetei [38] also found that living in rural areas was a stigma marker related to adolescent cannabis use. What cannot be ignored is that levels of access to mental health care services are vastly different between urban and rural residents [39]. People living in rural areas showed lower mental health literacy than people living in urban areas [40]. The study confirmed that the important issue of improving health literacy in rural areas in China is urgent and challenging. Similar with low education and low income students, digital video interventions may be a proper way for rural students to improve stigma [36].

In our study, a single marital status was associated with a positive attitude toward mental disorders. This result was inconsistent with another study showing that married people have a more tolerant attitude toward people with mental disorders than single people [41]. However, marital status had no significant relationship with attitude toward people with mental illness among medical students in Oman [42]. The discrepancy is interesting and worthy of further exploration. Although marriage is beneficial to people in promoting them to progressively accept different and unique people [41], it cannot explain the relationship between marital status and stigma in our study. A possible reason may be that participants were younger in our study, resulting in a shorter marriage experience. Reviewing previous literature, few studies have focused on the relationship between marital status and stigma in medical and nursing students. In our study, the number of married students was small. In the future, researchers could recruit more married students to confirm the relationship between marital status and stigma.

Similar to a previous study, medical and nursing students’ stigma was related to familiarity with people with mental disorders. Compared with students who had suffered from mental disorders, students who had not presented higher stigma. Compared with students who had a family member, a relative or a friend experiencing mental disorders students who had not showed a higher stigma. This result agreed with our earlier observations, which showed that people with higher familiarity were related to less stigma [43, 44]. However, a few studies have pointed out that more familiarity is positively associated with a higher level of stigma [45, 46]. A paper that reviewed previous literature pointed out a new opinion that familiarity is associated with stigma in a U-shaped curve [47]. According to this paper, in the low range of familiarity, familiarity was negatively associated with stigma, but once familiarity increased over an inflection point, familiarity was positively associated with stigma [47]. These results suggested that stigma may decline or increase with increased the familiarity with mental disorders or people with mental disorders. Therefore, for one hand increasing contact may not have a significant impact on students’ stigma for students with high familiarity, such as with a history of mental disorders or children of people with mental disorders. They could take part in self-help interventions to reduce stigma, such as web-based cognitive behavioral therapy and telephone tracking [48]. For another hand, clinical rotations or clinical training course may be beneficial for students with low familiarity with mental disorders, even when the training does not include any anti-stigma input [49].

### Limitations

The study has several limitations. The participants were recruited by convenience sampling online. There may be a selective bias resulting from the nonresponse of students with heavy stigma toward mental disorders or students with mental disorders. Moreover, the majority of the sample was female students and college or below students. Therefore, the current sample may not reflect the reality of medical and medical students. Random sampling and the inclusion of more male students will be considered in future studies. Another limitation is that the study is a cross-sectional study, which cannot determine the protective or risk factors for stigma toward mental disorders. Last, the study only focused on sociodemographic characteristics and familiarity with people with mental disorders as potential factors of stigma toward mental disorders. In future studies, researchers could explore more factors of stigma in medical and nursing students, such as whether they have an experience of rotation or internship in psychiatric departments.

## Conclusions

The large cross-sectional sample provides significant evidence of the outcomes and factors of stigma toward mental disorders in medical and nursing students in China. Medical and nursing students also show a negative attitude toward mental illness to a certain degree. Medical and nursing students hold an especially negative view that people with mental disorders are inferior. Most importantly, student stigma is significantly associated with student education, area of residence, marital status, economic status, and familiarity with people with mental disorders. Besides the regular Psychiatry, college or university could provide digital video as an understandable and accessible way to enhance mental health literacy and increase positive attitudes among rural, low education level, low income students. Clinical rotations or clinical training course may be beneficial for medical and nursing students with low familiarity with mental disorders to improve stigma toward mental disorders. Although the study enhances the literature on medical students’ stigma, more studies should explore the controversial factors of stigma to improve stigma in medical students.

## Data Availability

The authors confirm that the data supporting the findings of this study are available within the article. The corresponding author may be contacted for further data sharing: Ya Wang, 147493818@qq.com.

## Abbreviation

CAMI: Community Attitudes toward Mental Illness Scale
